# Editorial: Advances and Perspectives in Farm Animal Learning and Cognition

**DOI:** 10.3389/fvets.2019.00172

**Published:** 2019-06-04

**Authors:** Christian Nawroth, Jan Langbein

**Affiliations:** Institute of Behavioural Physiology, Leibniz Institute for Farm Animal Biology, Dummerstorf, Germany

**Keywords:** animal welfare, animal behavior, livestock, human-animal interactions, enrichment

The welfare of farmed animals is of major concern for society and food production (1–3). Of increasing relevance for understanding welfare is the knowledge on how farm animals perceive and deal with their physical and social environment. This information is crucial for applied ethology as it allows management practices to be adjusted to suit the animals' specific behavior and needs. The current Research Topic comprises 10 articles presenting state of the art research on farm animal learning and cognition. It includes novel and innovative empirical research, highlights the current state of farm animal cognition as well as its limitations, and discusses findings considering future interdisciplinary approaches and applications.

Three review articles summarize our present knowledge of different aspects of learning and cognition in farm animals and critically discuss their interpretation and potential for implementation. Nawroth et al. reports on the existing research on cognitive abilities of ungulate livestock species, focusing on a distinct set of cognitive capacities in the physical and social domain. They conclude that while research on livestock species is still underrepresented, the current findings indicate that ungulate livestock possess sophisticated mental capacities. They emphasized the importance of gaining a better understanding of how livestock species interact with their physical and social environments, as this information can be applied to improve housing and management conditions and to evaluate the use and treatment of animals in farming systems. From an ethical perspective, they also discuss whether animal cognition and the connection to animal welfare matters from the perspective of the animal.

Rørvang et al. critically evaluated the evidence for social learning in horses and the learning mechanisms involved. They conclude that many reported findings for social learning can be explained by relatively simple mechanisms such as social facilitation or stimulus and local enhancement, rather than by more complex phenomena such as emulation or true imitation (4). They state that, to date, there is no convincing evidence for true social learning in horses and discuss why the attribution of high-level social-learning abilities may even be maladaptive in horses.

A wide range of cognitive tests have been adapted or developed for the use in farm animals. The focus of most studies has been on variation of test performance at the species level; between- and within-individual differences of the same species remain largely unexplored. Bushby et al. summarized the contribution of factors such as choice of cognitive test, sex, early life environment, rearing conditions or personality to individual variation in cognitive outcomes. Further, the impact of such factors in recent farm animal studies is presented together with a framework on how to account for them statistically with special focus on experimental design and analytical techniques.

Inter-individual differences (5) in learning and cognitive performance of farm animals have also been a topic of strong interest in most of the five empirical studies that are covered in this Research Topic. Differences arising during early ontogeny, such as via differences in birth weight, were examined by Roelofs et al. They report the effects of low birth weight of piglets on post-weaning reference and working memory as well as learning flexibility. Their results show that pigs with low birth weight show a slightly impaired cognitive performance which goes along with higher long-term stress level in these individuals in comparison to pigs with normal birth weight.

Another factor rarely addressed in the investigation of individual differences in cognitive performance of farm animals is the prenatal stress to which mothers are exposed. To address this issue, Vas et al. examined how stress in pregnant goats, induced by reduced space allowance, affects their offspring's performance in a test on object permanence. In contrast to their initial hypothesis, they found that a higher prenatal maternal cortisol level was correlated with better performance by offspring on the most difficult task of the test.

Other cognitive traits might be important to consider, too, when talking about individual differences. The relationship between impulsive behavior and aggression in humans and animals might also have important implications for farm animal husbandry and welfare. Zebunke et al. adapted the “Marshmallow Test” to study impulse control in pigs. They found that piglets show impulse control, and that this is most strongly shown for rewards differing in quality rather than quantity. A broader understanding of impulse control might help in adapting husbandry conditions to the needs of individuals, especially in relation to social behavior, tail biting, and maternal behavior.

Intense selection for production traits is another factor that might account for differences in learning and cognition of farm animals. Dudde et al. investigated how laying performance and phylogenetic origin affect learning performance in laying hens. They hypothesized that there might be a trade-off between egg yield and cognitive performance in terms of the energy which is available. In contrast to their initial hypothesis, their results indicate that high performing laying hens performed better in a visual discrimination task compared to moderate productive hens in a feeding-rewarding context.

Another emerging area of research is how emotion and cognition are intertwined when it comes to interacting with conspecifics. Bellegarde et al. investigated if sheep can perceive the emotional valence displayed on the faces of conspecifics and how this valence affects their ability to discriminate between images of the same individual in different emotional contexts. They showed that sheep were able to differentiate between different emotional expressions of other sheep.

Two perspective articles outline future directions and potential implementations of basic research findings regarding the cognitive capacities of farm animals. Baciadonna et al. referred to the actual debate on how positive and negative emotions might spread by emotional contagion in farm animals (6) and argue in favor of future research on the mechanisms of how emotions in livestock are shared and how to use empathic responses to promote better welfare.

Finally, Lee et al. showcased how to take basic research into an applied setting. They developed a framework based on the cognitive activation theory of stress (CATS) and the cognitive evaluation of the environment in terms of predictability and controllability by an animal and used it in a case study to determine welfare outcomes of new technologies, here virtual fencing.

In conclusion, the contributions in this Research Topic will increase our understanding of how farm animals use their cognitive abilities to interact with their environment and aim to pave the way for new cross-disciplinary endeavors.

## Author Contributions

All authors listed have made a substantial, direct and intellectual contribution to the work, and approved it for publication.

### Conflict of Interest Statement

The authors declare that the research was conducted in the absence of any commercial or financial relationships that could be construed as a potential conflict of interest.
